# Taste and Smell Disorders in Cancer Treatment: Results from an Integrative Rapid Systematic Review

**DOI:** 10.3390/ijms24032538

**Published:** 2023-01-28

**Authors:** Tania Buttiron Webber, Irene Maria Briata, Andrea DeCensi, Isabella Cevasco, Laura Paleari

**Affiliations:** 1 Division of Medical Oncology, E.O. Galliera Hospital, 16128 Genoa, Italy; 2 Wolfson Institute of Preventive Medicine, Queen Mary University of London, London E1 4NS, UK; 3 Department of Health Professions, E.O. Galliera Hospital, 16128 Genoa, Italy; 4 Research, Innovation and HTA Unit, (A.Li.Sa.) Liguria Health Authority, 16121 Genoa, Italy

**Keywords:** dysgeusia, dysosmia, taste, smell, cancer treatment, rapid review

## Abstract

Taste and smell disorders (TSDs) are common side effects in patients undergoing cancer treatments. Knowing which treatments specifically cause them is crucial to improve patients’ quality of life. This review looked at the oncological treatments that cause taste and smell alterations and their time of onset. We performed an integrative rapid review. The PubMed, PROSPERO, and Web of Science databases were searched in November 2022. The article screening and study selection were conducted independently by two reviewers. Data were analyzed narratively. Fourteen studies met the inclusion criteria and were included. A high heterogeneity was detected. Taste disorders ranged between 17 and 86%, while dysosmia ranged between 8 and 45%. Docetaxel, paclitaxel, nab-paclitaxel, capecitabine, cyclophosphamide, epirubicin, anthracyclines, and oral 5-FU analogues were found to be the drugs most frequently associated with TSDs. This review identifies the cancer treatments that mainly lead to taste and smell changes and provides evidence for wider studies, including those focusing on prevention. Further studies are warranted to make conclusive indication possible.

## 1. Introduction

Global cancer statistics estimate that around 19.3 million new cancer diagnoses occurred in 2020. Lung cancer remained the leading cause of death, with an estimated 1.8 million deaths (18%), followed by colorectal (9.4%), liver (8.3%), stomach (7.7%), and female breast (6.9%) cancers [1].

Taste and smell disorders (TSD) are common side effects in cancer patients undergoing chemotherapy (CT) treatments but often are described as single entities and patients may have difficulty in identifying them [2]. The reported prevalence of taste disturbance ranged from 20% to 86% [3], and its development occurs approximately 2–3 weeks after the start of cancer treatment and persists throughout the duration of the therapy [4]. The prevalence of patients with dysosmia is in the range of 5–60% [5].

Interestingly, in the literature, it is reported that only a few patients report taste and smell alterations spontaneously and these symptoms are often underestimated by oncologists and nurses [6]. A study by Gill et al. reported a discrepancy in the importance given to retaining a normal sense of taste and smell, as reported by patients and by the multidisciplinary team involved in their care (*p* < 0.013) [7]. We previously hypothesized that the hesitancy of physicians in approaching these disorders may be due to a “cultural aspect” where the physician tends to underestimate and leave untreated the adverse events (AEs) related to therapies that do not have a clinical implication [8]. However, it is important to consider these disorders as they can lead to reduced food enjoyment and, most importantly, an inappropriate nutrient intake, with a high impact on the nutritional status, quality of life, and possibly on the efficacy of therapy itself [9].

Literature describes five basic tastes: sweet, sour, bitter, salty, and umami [10,11]. The sense of taste starts with the activation of the taste receptors located on the microvilli or taste receptor cells. These cells are clustered and together they form taste buds. Taste receptor cells are modified epithelial cells that can detect and process gustatory, olfactory, and trigeminal stimulation [12]. Dysgeusia can be classified as follows: (1) ageusia, which is a complete lack of taste; (2) hypogeusia, which is a decreased taste sensitivity; (3) hypergeusia, which is a heightened taste sensitivity; and (4) phantageusia, which is the perception of an unpleasant taste in the absence of a corresponding stimulus in the environment [9]. The sense of smell is even more complex than the sense of taste. Over a trillion different smells can be identified. There are two ways for odors to reach the olfactory epithelium: via the ortho-nasal passage or via the retro-nasal passage [13]. In general, four categories of smell disorders are classified depending on how they impact odor perception. (1) Anosmia is the absence of smell perception; (2) hyposmia is a quantitatively reduced ability to perceive scent; (3) parosmia is a qualitative distortion of an ordinarily detected smell; and (4) phantosmia is the perception of odors when none are present [14].

In addition to cancer treatment, including radiotherapy and surgical treatments, other factors may contribute to taste and smell disorders, such as age, oral infections, smoking, alcohol abuse, chronic diseases such as diabetes, hypertension, and chronic rhinosinusitis, and the type of cancer [15,16,17]. In fact, the study by Dhuibhir and colleagues showed a high prevalence of taste and smell disorders in newly diagnosed cancer patients before treatment [18].

Currently, TSD can be assessed through clinical methods (objective) or self-reported by patients (subjective). The objective methods assess the oral sensitivity to taste agents through the thresholds of the five taste qualities. The numerical results facilitate the comparison of the taste perception abilities between populations [19] but do not reflect the ‘real-world’ taste experience [20] as they do not capture dimensions of taste that are important to patients, such as flavor, food enjoyment, or hedonic changes [21]. For this reason, patient-reported questionnaires and qualitative research methods that capture patients’ individual experience are recommended [6,22].

A review by Enriquez-Fernandez et al. [23] reports a growing interest in the assessment of taste and smell changes in cancer patients but presents limitations in terms of the heterogeneity in the number of items, assessment range, and in the domains of taste changes. They suggest developing a standardized tool validated by patients to ensure that the terms associated with sensory changes are understood and reliably used by clinicians and researchers.

The aforementioned papers have mainly highlighted the pathophysiology, prevalence, clinical features, and assessment tools of chemosensory alterations. However, there are limited literature reviews highlighting the oncological therapies that lead to these alterations. The aim of this rapid review was to examine the existing and current literature on cancer treatments that can cause TSDs to develop prevention and education strategies in the future.

## 2. Materials and Methods

### 2.1. Study Design

Between October and November 2022, an integrative rapid review was conducted as a knowledge synthesis strategy to provide timely information [24]. Despite being thoroughly studied, the field of TSD is evolving due to novel cancer treatments. As a result, timely reviews can describe current research and report on clinical and organizational levels [25].

### 2.2. Needs Assessment and Topic Selection

The primary need was to map the most recent data on cancer drugs that cause TSD, to summarize the knowledge and enable nurses and oncologists to continuously improve the quality of care and patient management. Thus, the review question was: which oncological drugs cause taste and smell disorders? To this review, we decided to include only studies focused on cancer treatment for solid tumors in adults.

### 2.3. Study Development

According to the methodological process inspired firstly by Tricco and colleagues in 2017 [25], which was then further developed by Langlois et al. in 2019 [26], the following seven-stage process was implemented: (1) a needs assessment and topic selection; (2) study development; (3) literature search; (4) screening and study selection; (5) data extraction; and (6) risk-of-bias assessment. In addition, the Preferred Reporting Items for Systematic reviews and Meta-Analysis (PRISMA) guidelines were applied [27].

### 2.4. Literature Search

The literature search was performed independently by 2 reviewers (TBW and IMB) through the MEDLINE (via PubMed), PROSPERO, and Web of Science databases between October and November 2022. The inclusion criteria were: (a) studies on adult patients with solid tumors undergoing treatment with oncological drugs, (b) studies designed to detect the incidence and prevalence of TSD and/or assess the time of onset; (c) quantitative and qualitative primary studies; and (d) studies published in English within the past 10 years. The exclusion criteria were: (a) studies on patients with hematologic malignancies and (b) studies on patients undergoing radiation therapy. Therefore, the Medical Subject Headings [MeSH] and free-text words used were: “dysgeusia”, “taste alteration”, “anosmia”, “olfaction disorders”, “smell alteration”, “therapeutics”, “therapies”, “treatments”, “cancer”, and “neoplasm”. The research was limited to the last 10 years.

### 2.5. Screening and Study Selection

**Figure 1:**
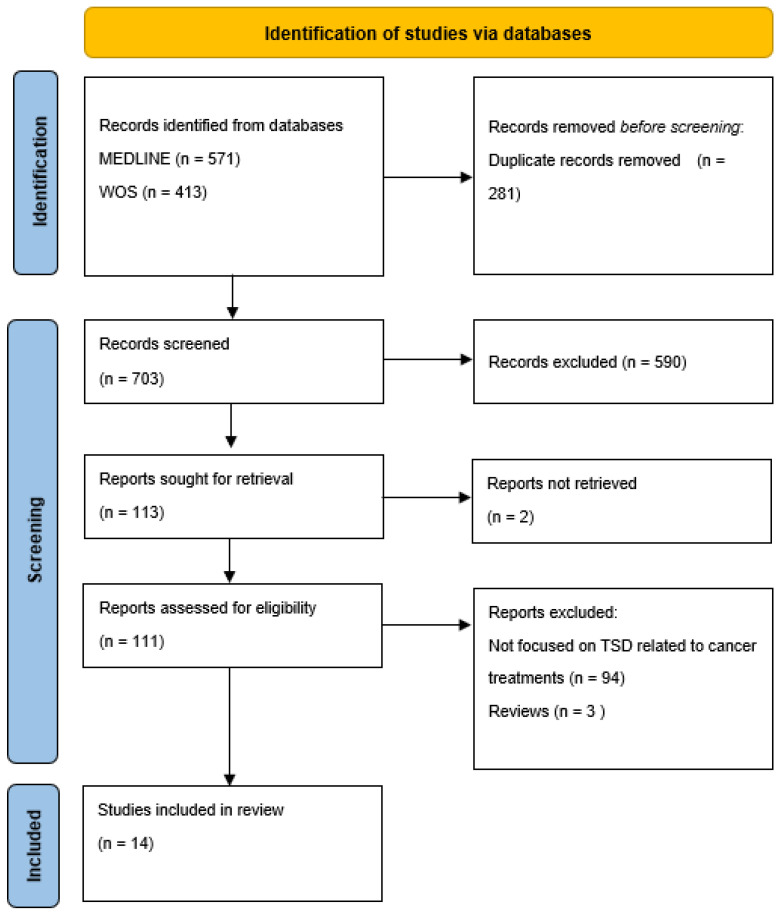
Flowchart of the data collection and selection process in accordance with PRISMA-ScR guidelines.

The screening of titles and abstracts was performed by three researchers (TBW, IMB, and LP) to identify the articles’ eligibility in relation to the inclusion criteria. Then, an independent full-text evaluation was performed by the same researchers to determine if the studies fully met the inclusion criteria. When disagreements occurred, the final decision to include or exclude an article was made by a consensus. As reported in the PRISMA flow diagram (Figure 1), the flow of a study’s inclusion is summarized together with the reasons for exclusion. At the end of the process, 14 papers were retrieved.

### 2.6. Data Extraction

The data were extracted and reported in a Microsoft Excel^®^ spreadsheet. The following data were collected from each selected study and reported in the grid: the (a) author(s), year, country; (b) study design; (c) aims; (d) participants; (e) assessment tool used for TSD detection; (f) cancer treatment; and (g) key findings. The full grid is available as Table 1.

### 2.7. Risk of Bias Assessment

The review team shared in advance the decisions about inclusion and exclusion to prevent information and a selection bias. In addition, to ensure the consistency of the results, the following methodological requirements [28] were respected: (a) the verification of the study selection and data extraction were performed by three reviewers [TBW, IMB, LP]) and (b) an additional independent researcher (=a fourth reviewer [ADC]) contributed and reviewed the narrative synthesis and the summary table.

**Table 1:** Characteristics of studies that evaluated cancer therapy-related dysgeusia.

Ref. n.	Study Design	Aim (s)	Participants (N)	Assessment Tool (s)	Cancer Treatments	Key Findings
[9]	Prospective	Prevalence	75 Prostate cancer	Survey regarding the taste and smell of food, appetite, and nausea.	CT and/or HT (regimen not specified)	TAs: 17%; SAs: 8%. TAs most frequent in patients treated with denosumab (35.0% vs. 10.9%, OR = 4.40, *p* = 0.020) or docetaxel (41.7% vs. 12.7%, OR = 4.91, *p* = 0.022). Poor taste of food associated with poor appetite and ≥10% weight loss.
[29]	Prospective	Self-reported TSDs based on the type of CT treatment. Impact of CT on the severity of the TSDs.	151	Questionnaire structured in three sections: eating habits; sensory changes (taste/smell changes and thermal sensitivity); and other clinical disorders (nausea, vomiting, dry mouth, mucositis, and dysphagia).	CT (regimen, Paclitaxel, oxaliplatin, docetaxel, carboplatin, anthracyclines, cisplatin, irinotecan, 5-FU, vinorelbine)	TAs: 76%. SAs: 45%. TAs in patients treated with anthracyclines, paclitaxel, carboplatin, and docet-axel. Cisplatin and 5-FU are the CT resulting in the lowest complaints. Xerostomia is strongly associated with bad taste in mouth (OR = 5.96; CI = 2.37–14.94; *p* value = 0.000) and taste loss (OR = 5.96; CI = 2.37–14.94; *p* value = 0.000).
[30]	Cross-sectional	Prevalence, severity and self-reported characteristics of TAs induced by CT. TAs across CT regimes.	243	Validated TA Scale Self-reported TAs duration CiTAS.	CT (regimen: FOLFOX, paclitaxel, docetaxel, cisplatin, pemetrexed, FEC, EC, FOLFIRI, Gemcitabina, TJ, TPF, Gemcarbo, Cisgem, Gemox)	TAs ranged from 51–86%. 43% of participants complained of TAs with the start of Ct and 75% reported TAs within the fourth week of treatment. TAs in patients treated with gemcitabine, cisplatin plus pemetrexed and epirubi-cin plus cyclophospha-mide (EC). Low levels of TAs were found among participants receiving GEMCARBO and CISGEM. 55% of participants reported some difficulties in tasting food. Tasting saltiness was the most affected ability.
[31]	Prospective	Incidence of TAs	41 BC	Not validated Questionnaire, filter paper disk method, CTCAE v. 4.0.	CT (regimen: Epirubicin, cyclophosphamide)	TA on the 4th day after CT was 53%. TAs decreased to about 9% immediately before new cycles. Age and body surface area influenced Tas.
[32]	Prospective	Prevalence TAs across CT regimes.	109 BC, gynecological	Validated TAs scale.	CT (regimen, Gemcitabina, epirubicin, docetaxel, capecitabine, epirubicin/docetaxel)	TAs was 76.1%. The highest TAs with epiru-bicin, docetaxel, capecita-bine. Lowest TAs with gem-citabine.
[33]	Prospective	To provide new data about TSDs.	33 head/neck	Sniffin’ Sticks test (Determination of threshold, discrimination, and identification, TDI).	CT (regimen: Cisplatin, carboplatin, 5-FU, docetaxel)	In normosmic or hyposmic, the mean decrease in TDI-score was significant lower during the second cycle. Age (>55 years) and smokers had a significant (negative) impact.
[34]	Prospective	Prevalence of dysgeusia.	31 males 15 females (9 did not undergo CT)	Salt-impregnated taste strips with 6 concentrations of Sodium chloride.	CT (regimen: 5-FU, platinum, Tx)	TAs in 38.8%. 48% in 5-FU or its oral analogues. 55.6% of patients receiving oral 5-FU analogues. Patients aged ≥70 years also tended to experience dysgeusia (75%).
[35]	Prospective	Effect of cisplatin CT on odor perception.	15 bronchial cancer patients and 15 control subjects	European Test of Olfactory Capabilities (ETOC).	CT (regimen: cisplatin)	Odor detection and odor identification abilities were not influenced by the administration of cisplatin, a decrease in pleasantness was observed only for food odors, and not for non-food odors.
[36]	Cross-sectional	TAs characteristics	100	Taste recognition thresholds (TRTs) via a taste disc kit PRO-CTCAE CiTAS.	CT (regimen: Tx based)	TAs in 59%. DTX associated with a higher prevalence of more severe and longer TAs than PTX or nab-PTX regimens. Significantly elevated taste recognition thresholds (hypogeusia) for sweet, sour, and bitter tastes in the taste alteration group receiving nab-paclitaxel (*p* = 0.022, 0.020, and 0.039, respectively). Docetaxel, previous CT, dry mouth, and peripheral neuropathy were significantly associated with Tas.
[37]	Observational	Prevalence and clinical therapeutic risk factors.	7425	CTCAE v5.0	CT (not specified)	TAs in 19.0%; 15.0% grade 1 dysgeusia and 6.0% grade 2. CT duration (*p* < 0.001), female sex (*p* < 0.001), location of the primary tumor in the uterus (*p* = 0.008), head and neck (*p* = 0.012), and testicles (*p* = 0.011), and use of ifosfamide (*p* = 0.009), docetaxel (*p* = 0.001), paclitaxel (*p* < 0.001), pertuzumab (*p* = 0.005), bevacizumab (*p* < 0.001), and dacarbazine (*p* = 0.002) independently increased the risk of dysgeusia.
[38]	Mixed methods	To investigate whether mycotoxic and/or neurotoxic drugs compromise olfactory performance.	44	Sniffin’ Sticks test (Determination of threshold, discrimination, and identification, TDI).	CT (regimen: Oxaliplatin, 5-FU, capecitabine, gemcitabine, carboplatin, cisplatin, doxorubicin, liposomal doxorubicin, taxanes)	TDI scores were significantly lower after chemotherapy in all age groups. Patients older than 50 years were more susceptible to olfactory toxicity.
[39]	Case control	Changes in the perception of tastes.	43	Taste strips	CT (regimen: platinum based)	Salty and sour were the most affected tastes in the study group (*p* = 0.001 and 0.05).
[40]	Observational	Changes in the detection (DT) and recognition (RT) thresholds of umami, sweet, and bitter tastes.	40 (NSCLC)	Rinsing technique. Not validated Questionnaire	CT (regimen: Cisplatin, paclitaxel)	TAs 34% after treatment. 42% reported a bitter taste in the mouth. 57% reported dry mouth. 35% reported food being tasteless, and 12% reported food having an unpleasant taste.
[41]	Qualitative	Patient and carer descriptions, experiences and consequences of taste and flavor changes.	10 patients 4 carers	Semi-structured interviews	Ct (regimen: Oxaliplatin)	TAs were apparent by the third CT cycle. Worse symptoms in the 5–7 days immediately post CT infusion, relief toward the end of a CT cycle. Full resolution of symp-toms by 6–8 weeks fol-lowing the completion of oxaliplatin treatment. Most common oral sensations: ‘metallic’ or a ‘slick’ or ‘coating’ in the mouth.

Abbreviations. CT: chemotherapy; HT: hormone therapy; 5-FU: 5-fluorouracil; TAs: taste alterations; FOLFOX: folinic acid, fluorouracil, oxaliplatin; FEC: epirubicin, fluorouracil, cyclophosphamide; EC: epirubicin, cyclophosphamide; FOLFIRI: folinic acid, fluorouracil, irinotecan; TA: taste alteration; TJ: carboplatin, paclitaxel; TPF: docetaxel, cisplatin, fluorouracil; GEMCARBO: gemcitabine, carboplatin; GEMOX: gemcitabine, oxaliplatin; CISGEM: cisplatin, gemcitabine; BC: breast cancer; CTCAE: Common Terminology Criteria for Adverse Events; Tx: taxane; CiTAS: Chemotherapy induced Taste Alteration Scale; PRO-CTCAE: patient-reported outcome; DTX: docetaxel; PTX: paclitaxel; nab-PTX: nab-paclitaxel; NSCLC: non-small cell lung cancer.

## 3. Results

### 3.1. Study Characteristics

A preliminary search aimed at exploring knowledge on cancer treatments causing TSDs was conducted by examining the MEDLINE database (via PubMed), Web of Science, and PROSPERO between October and November 2022. There were not recently published or ongoing reviews on this specific topic; meanwhile, a large number of articles on TSDs in cancer patients related to eating habits and quality of life have been published in recent years. The database searches, after the duplicates were removed, returned 703 articles, of which 113 were screened. Out of the 113 articles assessed for their eligibility, 14 studies met the inclusion criteria (Figure 1). The specific characteristics of each selected study that met the inclusion criteria are presented in Table 1. Most of the selected studies used a quantitative method [9,29,30,31,32,33,34,35,36,37,38,39,40], and only one was a qualitative study [41]. Nine studies focused on changes in the taste [30,31,32,34,36,37,39,40,41], 3 focused on smell [33,35,38], and 2 focused on a combination of both [9,29]. The study population among the studies was very different in terms of the cancer diagnosis, stage, treatment, line of therapy, and sample size. Moreover, a high degree of heterogeneity in the tools to assess TSD was observed, even within the study itself. In fact, some studies used a subjective evaluation [9,29,30] while others used validated questionnaires (e.g., CITAS) [30,36] or standardized measurement scales such as CTCAE [31,36,37]. Two studies used the Sniffin’ Sticks test [33,38] while one study used taste recognition thresholds (TRTs) [36].

### 3.2. Prevalence, Onset, Resolution of Taste and Smell Disorders

Taste disorders were found in 17% to 86% of people and were linked to a poor appetite and a 10% weight loss [29,30]. Campagna et al. reported that 43% of participants complained of TSDs at the start of CT, and 75% reported it by the fourth week of treatment [30]. The worsening of symptoms occurs within 5–7 days immediately following the CT infusion and decreases by about 9% immediately before the next cycle [31]. The complete resolution of symptoms (e.g., from oxaliplatin) occurs within 6–8 weeks after the completion of treatment [31,41]. Xerostomia is strongly associated with a bad taste in the mouth (OR = 5.96; CI = 2.37–14.94; *p*-value = 0.000) and a loss of taste (OR = 5.96; CI = 2.37–14.94; *p*-value = 0.000) [29]. Salty and sour were the most affected tastes (*p* = 0.001 and 0.05, respectively) [40]. Body surface, smokers, and people over the age of 70 had a significant negative impact on taste and smell [31,33,34]. The duration of CT (*p* = 0.001), female gender (*p* = 0.001), and location of the primary tumor in the uterus (*p* = 0.008), head and neck (*p* = 0.012), and testicles (*p* = 0.011) independently increased the risk of dysgeusia [37]. The prevalence of olfactory disorders ranged from 8% to 45%; in normosmics or hyposmics, the mean decrease in the threshold determination, discrimination, and identification (TDI score) was significant during the second cycle of cancer treatment and smoking and being over 50 years old were risk factors for smell alterations [9,29,33].

### 3.3. Cancer Treatment

Docetaxel is the main drug related to the occurrence of TSDs and is associated with a higher prevalence of more severe and longer taste alterations than paclitaxel or nab paclitaxel [36]. Anthracyclines, carboplatin, epirubicin, cyclophosphamide, capecitabine, and cisplatin/pemetrexed are also frequently related to TSDs [9,30]. Low levels of taste alterations were found in gemcitabine/carboplatin (GEMCARBO) and cisplatin/gemcitabine (CISGEM) combinations [30], as well as in cisplatin and gemcitabine administered individually [32]. Additionally, 5-fluorouracil (5-FU) or its oral analogues showed a high prevalence of TAs [34]. Docetaxel, previous CT, dry mouth, and peripheral neuropathy were significantly associated with taste alterations [36]. Regarding the effect of cisplatin on odor, detection, and identification abilities were unaffected by an administration of cisplatin; a decrease in pleasantness was observed only for food odors and not for non-food odors [35].

## 4. Discussion

In addition to oncology drugs, the literature reports hundreds of drugs from all major therapeutic classes that have been clinically reported to cause unpleasant and altered taste sensations when administered alone or in combination with other drugs. These unpleasant sensations include metallic and bitter tastes, a partial or complete loss of taste, and distortions and perversions of taste [42].

As suggested in the review by Schiffman et al., there are a number of topics which are useful for understanding the biological basis of drug-induced taste disorders: (1) the interaction of drugs with taste receptors on the apical side of the tongue in the oral cavity; (2) genetic differences among patients that affect the taste perception of drugs; (3) taste sensations caused by injectable drugs; (4) drug interactions caused by the use of multiple drugs; and (5) potential biochemical causes.

It is also important to recognize which groups are most vulnerable to this alteration. These include (1) the elderly, who use a disproportionate number of drugs [43], (2) people with certain genetic polymorphisms related to the perception of a bitter taste [44], (3) people with a reduced drug clearance [45], and (4) people with a drug metabolism [46].

TSDs in cancer patients are an often underestimated and underreported problem that may result from the disease and/or its treatment; this is probably because physicians and nurses do not regularly use standardized taste tests to verify and validate drug-related taste disorders in patients. Additionally, many disorders of taste cannot be categorized according to conventional tastes such as sweet (sucrose), sour (citric acid), salty (NaCl), bitter (quinine), or umami (monosodium glutamate). Dysgeusia and dysosmia alter the pleasure of eating and reduce appetite, which, especially in compromised patients, can lead to malnutrition, increased treatment-related toxicities, and a worsened quality of life. Therefore, the identification of risk factors, such as the use of a specific oncological treatment, that may promote the development of TSDs, is an important aspect to reduce the impact of this condition on these frail patients. Our research identified 14 articles published in the last 10 years that investigated cancer treatments leading to TSDs. In accordance with the existing literature, the range of taste alterations varies between 17% and 86% [29,30] and the severity of the symptoms varies during the cycle [30,31,35]. In fact, the symptoms severity tends to worsen 5–7 days after the CT cycle and then diminish by about 10% before the following cycle [35]. Moreover, the taste alterations tend to persist for a long time [31,41], suggesting that the risk of malnutrition and a worsened quality of life may continue even after the end of the cancer treatment. Salty and sour tastes seem to be the tastes which are most affected by cancer treatments [40], so it might be useful to provide patients with specific nutritional guidance aimed to minimize the alterations. Dysosmia is less investigated but still its prevalence ranges from 8 to 45% of cancer patients [9,29]. According to the results of the current study, dysgeusia and dysosmia were more strongly associated with breast, gynecological, and colorectal cancer [32]. Docetaxel, paclitaxel, nab-paclitaxel, capecitabine, cyclophosphamide, epirubicin, anthracyclines, and oral 5-FU analogues were found to be the drugs most frequently associated with TSDs [9,30,34,36]. Other important risk factors for TSDs included the number of chemotherapy cycles, the female sex, the presence of distant metastases, and the primary tumor’s location in the uterus, testicles, or head and neck [37]. An interesting correlation emerged between dysgeusia and peripheral neuropathy; numbness or tingling in the hands or feet (OR, 2.04; 95% CI, 1.25–3.57; *p* = 0.004) were significantly associated with TAs [36]. Knowing the factors most associated with TSDs is crucial for physicians and nurses to carefully monitor their occurrence and severity and to implement adequate prevention strategies. Sevryugin et al., in a recent review [3], summarized a wide range of therapy alternatives, including zinc and polaprezinc, radioprotectors, vitamins and supplements, anti-xerostomia agents, active swallowing exercises, nutritional interventions, delta-9-tetrahydrocannabinol, and photobiomodulation that can be used as a strategy to reduce TSDs.

The high heterogeneity among the selected studies in terms of the diagnosis, stage of disease, treatment, the instruments used to assess the TSDs, and the sample size makes it difficult to make firm conclusions. The limited number of studies exploring specifically which cancer therapies cause alterations in taste and smell leads us to hypothesize that these disorders have not yet been given due attention. In addition, none of the studies included in the present review considered new therapies, such as immunotherapy, suggesting that further studies are needed to investigate the impact of cancer therapies more comprehensively on TSDs. Most published studies relate taste and smell alterations to quality of life, so interventions in a preventive context would be necessary; although, there is no consensus on the prevention strategies to be used in this setting, so an algorithm for selecting the best treatment for TSDs was developed [3]. The algorithm can help the clinician to provide a therapeutic solution for chemosensory disorders or it can help the researcher to design an appropriate clinical trial to increase the knowledge on this underestimated problem.

### 4.1. Study Limitations

This rapid review aimed to highlight current cancer drugs that can cause changes in taste and smell; however, we know that there are no studies that take into consideration other therapies such as hormone therapy, target therapies, immunotherapies, and monoclonal therapy. Furthermore, the wide heterogeneity of the evaluation tools used, and the different moments of detection do not allow for an accurate generalizability of the results. Our results are not to be considered conclusive, as another limitation is that we explored a limited number of databases.

### 4.2. Implications for Clinical Practice and Research

Oncologists and nurses should be trained on treatments that induce taste and smell disorders to educate patients about proper nutrition and reduce the impact of these symptoms on their quality of life.

## 5. Conclusions

Taste and smell disorders are not life-threatening events for patients but have a significant impact on their quality of life. Oncologists, nurses, and nutritionists play an important role in the management of these chemotherapy-related symptoms, so further studies are needed to provide specific information to patients on which oncology drugs cause dysgeusia or anosmia, the time of their onset and duration, and to support clinical governance strategies as well.
